# Complex Reporting of the COVID-19 Epidemic in the Czech Republic: Use of an Interactive Web-Based App in Practice

**DOI:** 10.2196/19367

**Published:** 2020-05-27

**Authors:** Martin Komenda, Vojtěch Bulhart, Matěj Karolyi, Jiří Jarkovský, Jan Mužík, Ondřej Májek, Lenka Šnajdrová, Petra Růžičková, Jarmila Rážová, Roman Prymula, Barbora Macková, Pavel Březovský, Jan Marounek, Vladimír Černý, Ladislav Dušek

**Affiliations:** 1 Institute of Biostatistics and Analyses Faculty of Medicine Masaryk University Brno Czech Republic; 2 Institute of Health Information and Statistics of the Czech Republic Prague Czech Republic; 3 Ministry of Health of the Czech Republic Prague Czech Republic; 4 National Institute of Public Health Prague Czech Republic; 5 Department of Anesthesiology Perioperative Medicine and Intensive Care Masaryk Hospital Ústí nad Labem Czech Republic; 6 Jan Evangelista Purkyne University Ústí nad Labem Czech Republic

**Keywords:** coronavirus disease, COVID-19, Czech Republic, web app, interactive reporting, epidemiological overview, CRISP-DM, public health, app, epidemiology, virus, health data, data mining, modeling

## Abstract

**Background:**

The beginning of the coronavirus disease (COVID-19) epidemic dates back to December 31, 2019, when the first cases were reported in the People’s Republic of China. In the Czech Republic, the first three cases of infection with the novel coronavirus were confirmed on March 1, 2020. The joint effort of state authorities and researchers gave rise to a unique team, which combines methodical knowledge of real-world processes with the know-how needed for effective processing, analysis, and online visualization of data.

**Objective:**

Due to an urgent need for a tool that presents important reports based on valid data sources, a team of government experts and researchers focused on the design and development of a web app intended to provide a regularly updated overview of COVID-19 epidemiology in the Czech Republic to the general population.

**Methods:**

The cross-industry standard process for data mining model was chosen for the complex solution of analytical processing and visualization of data that provides validated information on the COVID-19 epidemic across the Czech Republic. Great emphasis was put on the understanding and a correct implementation of all six steps (business understanding, data understanding, data preparation, modelling, evaluation, and deployment) needed in the process, including the infrastructure of a nationwide information system; the methodological setting of communication channels between all involved stakeholders; and data collection, processing, analysis, validation, and visualization.

**Results:**

The web-based overview of the current spread of COVID-19 in the Czech Republic has been developed as an online platform providing a set of outputs in the form of tables, graphs, and maps intended for the general public. On March 12, 2020, the first version of the web portal, containing fourteen overviews divided into five topical sections, was released. The web portal’s primary objective is to publish a well-arranged visualization and clear explanation of basic information consisting of the overall numbers of performed tests, confirmed cases of COVID-19, COVID-19-related deaths, the daily and cumulative overviews of people with a positive COVID-19 case, performed tests, location and country of infection of people with a positive COVID-19 case, hospitalizations of patients with COVID-19, and distribution of personal protective equipment.

**Conclusions:**

The online interactive overview of the current spread of COVID-19 in the Czech Republic was launched on March 11, 2020, and has immediately become the primary communication channel employed by the health care sector to present the current situation regarding the COVID-19 epidemic. This complex reporting of the COVID-19 epidemic in the Czech Republic also shows an effective way to interconnect knowledge held by various specialists, such as regional and national methodology experts (who report positive cases of the disease on a daily basis), with knowledge held by developers of central registries, analysts, developers of web apps, and leaders in the health care sector.

## Introduction

In early 2020, the pandemic of the coronavirus disease (COVID-19) started to spread all over the world. COVID-19 is caused by a novel type of coronavirus, referred to as severe acute respiratory syndrome coronavirus 2 (SARS-CoV-2). This highly infectious disease is mainly manifested by fever, respiratory difficulty (cough, dyspnea), muscle pain, and fatigue. The disease can be rather serious for people who are older or have chronic illnesses and can even be fatal. The beginning of the COVID-19 epidemic dates back to December 31, 2019, when the first cases were reported in the city of Wuhan in Hubei Province in the People’s Republic of China. Over the next 4 months, more than 1,120,000 people became infected across the world and almost 60,000 patients died from the disease [1]. In the Czech Republic, the first 3 cases of infection with the novel coronavirus were confirmed on March 1, 2020. A team of experts from the Institute of Health Information and Statistics of the Czech Republic (IHIS CR), together with researchers from the Institute of Biostatistics and Analyses at the Faculty of Medicine of the Masaryk University, focused on the design and development of a web app intended to provide a regularly updated overview of COVID-19 epidemiology in the Czech Republic to the general public. The joint effort of state authorities and researchers gave rise to a unique team, which combines methodical knowledge of real-world processes with the know-how needed for effective processing, analysis, and online visualization of data. The requirements on functionalities of this tool were mainly defined by the seriousness of an unexpected situation; an urgent need emerged for a tool that would make it possible to present important reports based on valid data sources only. To achieve this goal, it was necessary to ensure that individual graphs, maps, and tables would be easy to understand and could be correctly interpreted by the public and the media, and that misinterpretation of outputs would be avoided. An extensive review of tools available abroad was a valuable input for the development of a Czech tool. Worldwide, most papers published until recently [2-10] have been focused on the research of populations affected by COVID-19, on the structure of SARS-CoV-2 and its comparison with similar viruses (Middle East respiratory syndrome-related coronavirus and severe acute respiratory syndrome-related coronavirus), and on treatment and the overall mortality of COVID-19. From the technological point of view, several articles have been dedicated to the issue of data collection and sharing, together with their online presentation [1,11-15]. There are several examples of portals documenting the COVID-19 epidemiology clearly on a nationwide level, such as the Icelandic COVID-19 in Iceland – Statistics; the Korean Coronavirus Disease-19, Republic of Korea; or the Singaporean Dashboard of the COVID-19 Virus Outbreak in Singapore.

The aim of the research team was to adopt one of the time-tested methodologies for data mining, analytics, knowledge discovery, and data science projects, and to apply it in the process of mapping the current COVID-19 epidemic situation in the Czech Republic. This paper describes all essential steps from methodological as well as technical points of view. The Czech approach to the design, development, and implementation of online monitoring of the COVID-19 epidemic is based on a verified methodology for the acquisition, processing, and presentation of information. The methodology discussed in this paper made it possible to interconnect knowledge held by various specialists such as regional and national methodology experts from the National Institute of Public Health and regional public health authorities, who report positive cases of the disease on a daily basis, with knowledge held by developers of central registries, analysts, developers of web apps, and leaders in the health care sector.

## Methods

### Methodological Background for the COVID-19 App: An Overview of the Current Situation in the Czech Republic

When the urgent need emerged to map the current situation regarding the COVID-19 epidemic, it was essential to consider several factors that might have a significant impact on the resulting reports. The infrastructure of a nationwide information system run by the public administration is one of the most important factors in this regard; data from basic registries as well as data from health service providers are collected and processed in this information system. The cooperation between organizations involved in the process of data reporting, collection, processing, validation, analysis, evaluation, and visualization is another key aspect. The methodological setting of communication channels, duties resulting from legal measures, clearly defined competence, the sequence of steps to be made, and the overall management of the health care sector are complex at the time of an unexpected pandemic. In addition, it is important to select appropriate procedures for knowledge mining from database structures and for an undistorted interpretation of data provided to individual target groups. These groups involve not only the general public and health care professionals but also the media, as the resulting reports are published on behalf of the Ministry of Health of the Czech Republic and can, therefore, be considered as guaranteed and entirely reliable.

Among several known methodological recommendations and standardized procedures, which can be cited as the implementation of the knowledge discovery in databases, the process Sample, Explore, Modify, Model, Assess and cross-industry standard process for data mining (CRISP-DM) are used most frequently in practice [16]. The team of authors chose the latter methodology (CRISP-DM) due to its higher versatility. The individual stages of this methodology directly correspond to the complex solution of analytical processing and visualization of data, which provides validated information on the COVID-19 epidemic across the Czech Republic. The power of CRISP-DM is demonstrated by the fact that great emphasis is put on the understanding and on a correct implementation of all six steps needed in the process (see Multimedia Appendix 1); furthermore, CRISP-DM provides the option to return back to previous steps, and importantly, it does not leave out the frequently omitted process of checking the achieved results before their publication.

#### Business Understanding

**Figure 1:**
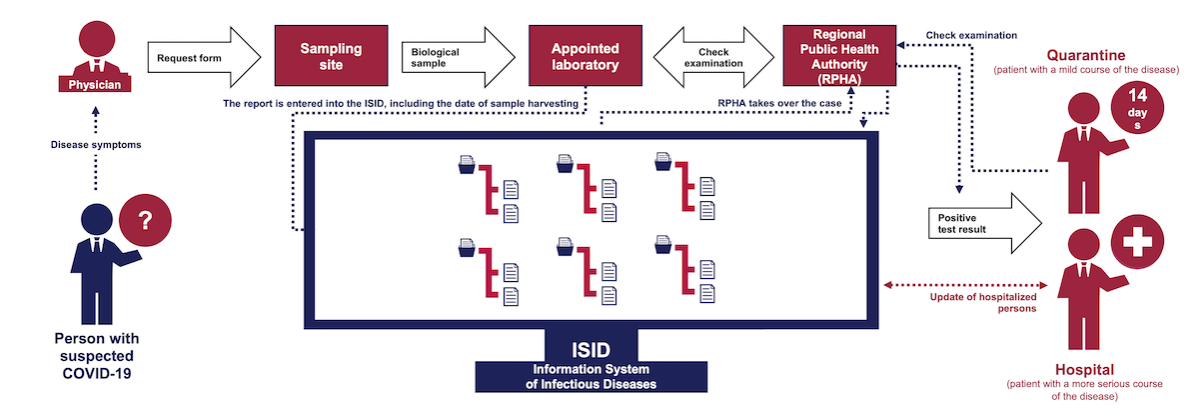
Simplified schema of newly identified cases of COVID-19. COVID-19: coronavirus disease.

In the initial stage, maximum attention is paid to mapping the situation from the managerial point of view. In this case, the task is focused on online visualization of data on the current state of the COVID-19 epidemic in the Czech Republic. The Ministry of Health of the Czech Republic is responsible for the methodological setting of regular reports and processing data on newly identified cases of COVID-19 across the Czech Republic (see Figure 1), record keeping on testing locations, and overviewing the purchases and distribution of personal protective equipment (PPE). The reporting process always starts with a person with suspected COVID-19 being referred by a physician or a regional public health authority (RPHA) to a testing location, where a biological sample is taken from them, in compliance with the RPHA methodology. The sample is then analyzed by one of the appointed laboratories, which determines whether or not it is positive for SARS-CoV-2. The result is subsequently entered into the central Information System of Infectious Diseases (ISID), which is then taken over by the respective RPHA. In the next step, the same RPHA carries out a second investigation to verify the result, informs the person about the result, and, if the sample was confirmed to be positive for SARS-CoV-2, provides information about the next steps to be taken. The systematically designed architecture of the National Health Information System (NHIS) made it possible for the result of each performed test to be processed by a laboratory and verified by the respective RPHA in the central ISID, both procedures being done with only a minimum delay. The primary objective of the ISID is to obtain information on the incidence of infectious diseases to assess the epidemiological situation across the Czech Republic, to monitor the population’s health status, and to control the provision of health care. In compliance with section 70, paragraph 3 of the Act No 372/2011 Coll, on Health Services and Conditions of Their Provision (Act on Health Services), the administration of NHIS has been delegated to the IHIS CR. NHIS is a fully computerized system involving components that are enshrined in the legislation. Each person in the Czech Republic can be unequivocally identified based on their birth certificate number; based on this number, essential links can be found between the NHIS and other relevant databases run by the public administration, such as the National Register of Hospitalized Patients, the Registry of Inhabitants, the National Register of Health Services Providers, the National Register of Health Care Professionals, or the Death Records Database. Data from these registries provide a comprehensive—and, most of all, up-to-date—data basis for subsequent analytical processing. On top of that, the unequivocal identification of patients, which is identical across these registries, made it possible to obtain information on the infection rate among health care workers in real time, for example.

#### Data Understanding

**Figure 2:**
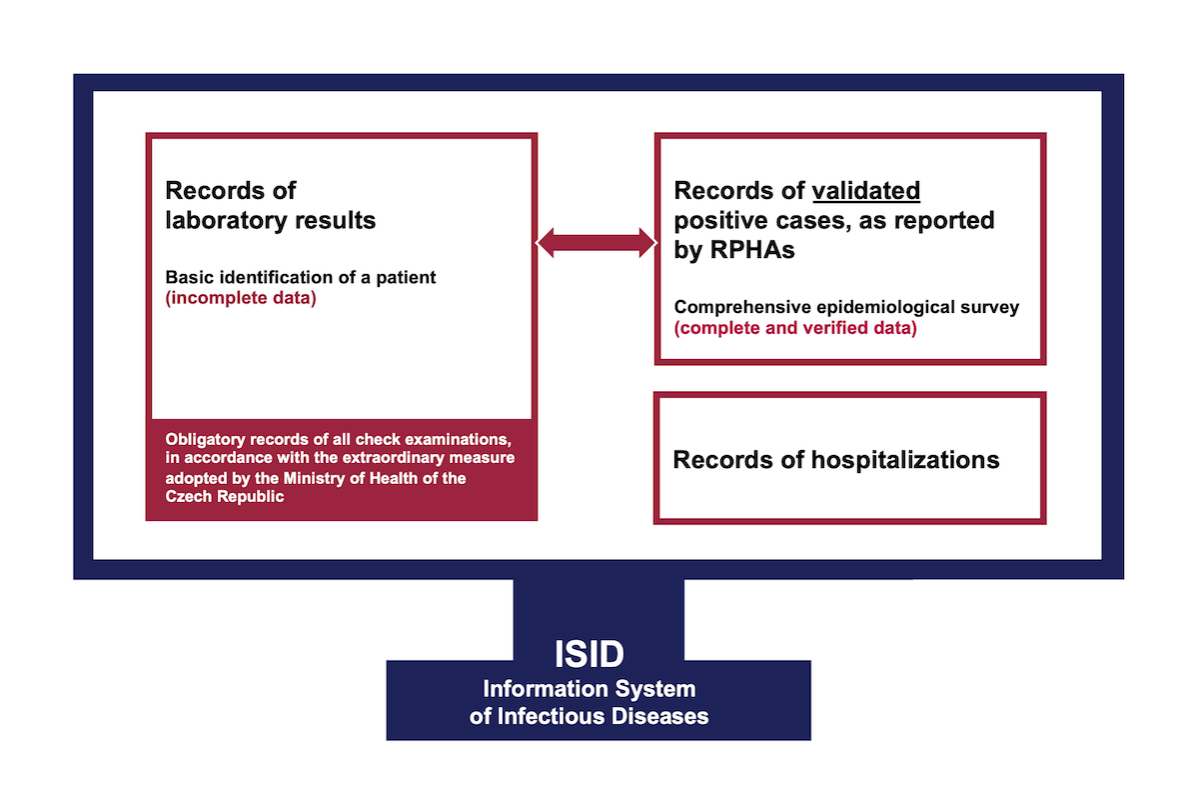
Diagram of data transfer within the Information System of Infectious Diseases.

For a correct collection and processing of information on the COVID-19 epidemiology in the Czech Republic, it is essential that all reporting processes are well understood, that communication with all involved institutions runs smoothly, that the architecture of central databases is well designed from the technical point of view, and that the resulting reports are correctly interpreted. At the same time, all interactive outputs, whether in the form of graphs, maps, or tables, which are available for the general public, must meet the condition that it will never lead to a direct identification of any patient. It is therefore not possible to show detailed information on a district (or even a town) in combination with sex or age of a given person. What can be published, on the other hand, are summary data on the entire Czech Republic (or possibly on individual regions or districts), as the population of the entire country (or region or district) is large enough and the total number of positive cases is higher than 10, which cannot lead to the direct identification of a particular patient. Other examples of outputs that can be published involve daily reports on cumulative numbers and on the increase or decrease in the number of confirmed cases or division according to sex or age groups. When processing personal data in the various part of NHIS, every individual record must be processed in accordance with Regulation (EU) 2016/679 of the European Parliament and of the Council of April 27, 2016, on the protection of natural persons with regard to the processing of personal data and on the free movement of such data, and repealing Directive 95/46/EC (General Data Protection Regulation), Act No 372/2011 Coll, on Health Services and Conditions of Their Provision (Act on Health Services), as subsequently amended, and the Act No 110/2019 Coll, on the Processing of Personal Data, as subsequently amended. The interactive online reporting is based on the five following data sets, which, combined together, characterize the COVID-19 epidemic in the Czech Republic. First, reports by RPHAs contain daily records of persons with confirmed COVID-19 (eg, date of report, age, sex, region, location, and country of infection). Second, reports by laboratories (LAB) contain deidentified records on persons with confirmed COVID-19, which have not yet been taken over by a RPHA in the respective region. In relation to reports by RPHAs, these are disjointed sets of records (each particular record goes to the LAB repository and consequently is either approved or directly confirmed by a respective RPHA); in other words, none of the records are present in both data sets, and undesirable duplicates are, therefore, avoided. Figure 2 shows how records provided by RPHAs and LABs are transferred into ISID. Among necessary adjustments, patient identification against the Registry of Inhabitants is performed at this stage, aiming to determine the region of a citizen who has been confirmed as a positive case by the laboratory. Third, a report on performed COVID-19 tests contains the number of all samples tested by laboratories across the Czech Republic on individual days. Fourth, reports on persons hospitalized in health care facilities contain daily summaries of currently hospitalized persons, persons in a serious condition or receiving highly intensive care (eg, mechanical ventilation, extracorporeal membrane oxygenation), and hospitalized persons who have been cured or discharged to home quarantine. Fifth, a report by the Ministry of Health of the Czech Republic contains purchases and distribution of PPE, and provides up-to-date numbers of face masks, respirators, goggles, bottles with disinfectant, face shields, and other equipment.

#### Data Preparation

**Figure 3:**
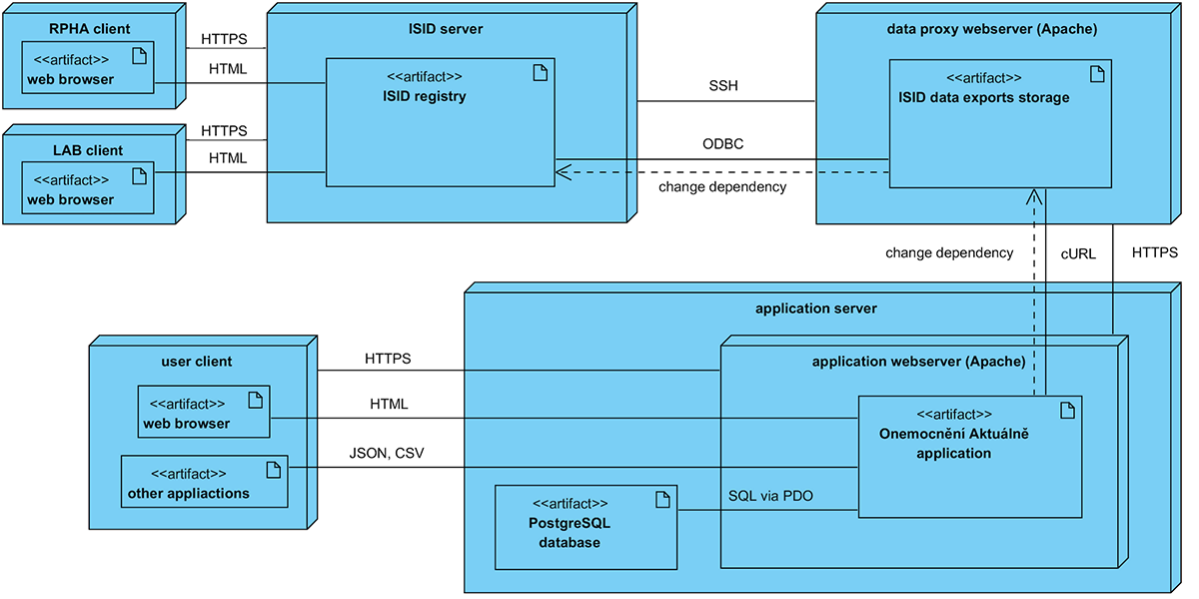
Deployment diagram. CSV: comma-separated values; cURL: Client URL; ISID: Information System of Infectious Diseases; JSON: JavaScript Object Notation; LAB: laboratories; ODBC: Open Database Connectivity; PDO: PHP Data Objects; RPHA: regional public health authority; SQL; Structured Query Language; SSH: Secure Shell.

Several different data sources are used as input at this stage; these data sources must be validated thoroughly and then transformed into a form suitable for computer processing and subsequent visualization. In particular, the validation process involves a set of control mechanisms ensuring the completeness of individual records. This stage involves data cleaning (incomplete records are detected, corrected, or removed), construction of particular data views (selected attributes with primary identification are derived and merged), and data integration (final data sets are automatically generated). This process ensures that no invalid records are included in the stage of further processing and web visualization. Syncing tests, which are launched at regular intervals, are among the main control mechanisms. These tests provide information on whether or not data presented in the end report are consistent with data on the input. Their objective is to detect possible inconsistencies in the input records, such as a patient’s incomplete or incorrectly entered birth certificate number; their permanent address; or distinction between a Czech citizen, a foreigner, or even a person who is homeless. If possible, standardized lists of values (ie, those valid on a nationwide level) are integrated, making it possible to name values in a unified way. As an example, a standardized classification of territorial units in the Czech Republic has been used (Nomenclature of Units for Territorial Statistics) based on unique codes for each of the 14 Czech regions (eg, CZ064 was used as a code for the South Moravian Region). Valid records in the ISID form the basis for the selection of descriptive attributes and their final adaptation into a format in which they are sent by export tools at regular intervals and in a secured manner to the web server. Figure 3 shows a diagram of implementation of individual components, showing the communication among three independent servers transmitting data sources that are necessary to draw the final visual outputs and to provide open data sets to the public.

#### Modelling

In this step, detailed static instructions were prepared for each report (graph, map, table), always involving its data source, computational algorithm, description of visualization, explanation of its meaning, and information on the last update. Afterward, the entire process of data processing and dynamic drawing of the online report was transformed into a fully automatic one. All predefined data sets are transferred into an internal data storage device, which is dedicated to epidemiological analyses and reporting; individual overviews are subsequently generated in the form of interactive graphs, maps, and data tables. The resulting presentation is a set of interactive graph visualizations and tabular outputs, which can be viewed online by anyone using only a standard web browser. The portal has been developed using the Symfony 4.4 PHP framework. Graphical outputs are processed by the NVD3 JavaScript library, which is based on d3.js. Graphs are slightly adjusted to meet the report’s needs, mainly in the responsive environment of the contemporary imaging technology. Tabular data are subsequently processed using the DataTables library, which internally employs jQuery (The jQuery Team). In this case, the data layer mainly consists of preprocessed and saved files in the JavaScript Object Notation (JSON) format. This approach significantly accelerates the access to data, making it possible to accelerate the app’s response markedly. On each update, data are transformed straight away, source files being replaced by new ones. The number of computational operations that are carried out at the app layer is kept at a minimum. The presentation layer deals with the graphical appearance of the user interface with control elements as well as the interactivity of the visualization. All reports prepared in this way are first implemented on development servers, where functionality and data correctness are thoroughly verified. Development servers are also used to check syncing with external data sources. Individual versions are subsequently published, always within the logical structure of the web app, which is divided into thematic sections. The app itself has been designed to be responsive and is fully supported by all types of devices, including the portable ones (mobile, tablet, and desktop).

#### Evaluation of Results

It is extremely important to validate the results before their publication, particularly when providing information about the spread of an epidemic. Despite several optimization and transformation processes performed in the preceding stages, data must remain consistent and all reports must correspond with the original input as well as with calculations carried out to check agreement with primary data. User testing not only revealed some inconsistency in original values obtained by static calculations, but also brought new ideas on how to improve the intelligibility, technical implementation, and user interface. Experts on accessibility of web apps were called in too. The target group was also taken into consideration, as it consists of disabled users, among others, for whom navigation on the internet might be difficult. A correct structure of headings, an adequate contrast between texts and the background, the overall legibility, a properly defined alternative information for images, and the availability of complementary tabular overviews next to graphs and maps are the most important accessibility attributes that have been thoroughly applied. One must keep in mind that web apps guaranteed by the state administration are required to provide maximum accessibility for all citizens without exception.

#### Deployment of Results

Primary data are entered into the ISID by LAB and RPHAs. After their validation and the unification of data formats, two data exports are created, and these are periodically sent from the ISID to a location from where they can be finally used for the purposes of the web portal. The periodicity of updates is set to 2 hours, with a nighttime shutdown between 2 am and 6 am The cron tool launches the updating script on the side of the “Disease at the Moment” (“Onemocnění aktuálně” in Czech) app at predefined times, which results in the transformation of provided data into preprocessed data sets. The previously mentioned syncing tests are launched during this process as well. The entire process is launched on the production instance of the “Disease at the Moment” app, as well as on the development instance and the stage instance. The latter two instances are automated by Jenkins, which is a continuous integration tool. In case of any error in data assembly or in the update of the entire app, an email notification is sent to the development team, allowing it to react appropriately. At the time of writing this paper (April 2020), primary data are updated three times a day on the production instance (12:30 am, 8:30 am, and 5:30 pm), and secondary data are usually updated once a day.

### Open Data for COVID-19

Science is built on data, namely their collection, analysis, publication, reanalysis, critique, and reuse. Barriers include inability to access data, restrictions on usage applied by publishers or data providers, and publication of data that are difficult to reuse, for example, because they are poorly annotated or ‘‘hidden’’ in unmodifiable tables like PDF documents [17]. For that reason, the concepts of open access and open data are strongly emphasized and play a key role in the complex web-based reporting of the COVID-19 epidemic in the Czech Republic. The Ministry of the Interior of the Czech Republic guarantees and maintains the National Catalogue of Open Data, which consists of 24 local instances, including the Catalogue of Open Data run by the Ministry of Health of the Czech Republic. The main goal is to collect metadata about data sets published as open data throughout the whole country and to show transparency and effectiveness of government services [18]. There are three major target groups of potential users who can access various data sets freely: (1) those who are interested in information describing the entire concept together with benefits of broad usage of open data sets; (2) those who want to publish and update various data sets in accordance with the given open data rules, such as diagrams, data formats, keywords, and metadata description; and (3) those who can browse and freely use available data sets for the purpose of further analysis and development. The authors of this paper decided to adopt a standardized methodology for the publication of open data, and thus facilitate the use of COVID-19 epidemiological data sets by different public sector bodies, academic institutions, and business sector companies.

### Google Analytics as the Monitoring Service

Monitoring and further analysis of web usage is one of the crucial points in terms of reflecting users’ behavior and requirements. Systematic tracking and web analysis significantly improve the efficiency and quality during a long-term design and development of robust web apps. Google Analytics is a third-party service that measures and generates up-to-date statistics, reports, and analyses based on website traffic and on the behavior of its visitors. It tracks the visitors’ activity, collects statistical data in real time, and stores them for a later analysis. Using different types of metrics, we can easily determine the number of visitors over any period, which pages they viewed, and how long was their visit. The flow of visitors is an important factor, showing user transitions between pages and the rate of abandonment of every single page [19]. Based on Google Analytics’ powerful features, advanced visualizations mapping the analytics intelligence, dashboard, mobile device tracking, referrers, and geographic tracking capabilities have been used [20].

## Results

### Description of the COVID-19 App: An Overview of the Current Situation in the Czech Republic

On March 11, 2020, the first version of the web portal was released [21]. It provides a set of outputs in the form of tables, graphs, and maps intended for the general public and the media. Its primary objective is to provide a well-arranged visualization and clear explanation of basic information included in the basic overview of COVID-19 epidemiology in the Czech Republic (see Multimedia Appendix 2). The overall numbers of performed tests, confirmed cases of COVID-19, persons who have recovered from the disease, and COVID-19-related deaths are displayed first to the user. The rest of the webpage is divided into several sections with coherent topics, each of them providing a synoptic overview from a selected perspective. First, basic overviews according to reports by RPHAs and positive results from laboratories show the overall number of persons who were tested positive for COVID-19, the incidence of confirmed cases of COVID-19 by region per 100,000 people, absolute numbers of people with positive COVID-19 cases by region, the overall number of performed tests (including repeated tests in the same person) for COVID-19 across the entire Czech Republic, and an overview of COVID-19-related deaths by age group and by region. Second, daily overviews according to reports by RPHAs and positive results from laboratories show a daily overview of number of persons with newly confirmed COVID-19, a daily overview of the number of performed tests (including repeated tests in the same person), the overall (cumulative) number of persons with laboratory-confirmed COVID-19 and the daily percentage change, and the daily percentage of persons with confirmed COVID-19 in the number of performed tests on a given day. Third, the number of persons with laboratory-confirmed COVID-19 according to reports by RPHAs shows an overview of cumulative numbers of persons with laboratory-confirmed COVID-19 that have been verified by RPHAs (not the number of all persons with laboratory-confirmed COVID-19), the location and country of infection of people with a positive COVID-19 case, and the number of persons with laboratory-confirmed COVID-19 by sex and age group. Fourth, an overview of hospitalizations of patients with COVID-19 shows the current number of hospitalized persons, the number of persons in a serious condition or receiving highly intensive care, and the number of hospitalized persons who have been cured or discharged to home quarantine. Fifth, an overview of the distribution of PPE shows how much PPE has been purchased and distributed by the Ministry of Health of the Czech Republic across individual regions.

Because published data are based on several independent sources, it is obvious that updates must be performed at different times. Nevertheless, fixed time intervals have been set at which updated values are presented on the web. The latest numbers summing up the incidence of COVID-19 in the Czech Republic are updated 3 times a day: at 12:30 am, at 8:30 am, and at 8:30 pm. The overviews showing total numbers from the previous day are prepared each morning at 8:30 am, and the latest numbers of recovered persons and COVID-19-related deaths are published at 8:30 am and at 5:30 pm.

### Types of Visualization

**Figure 4:**
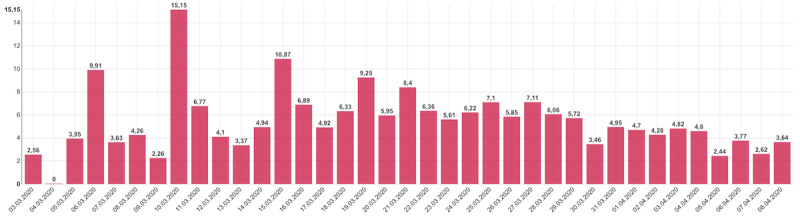
Daily trend in the percentage of persons with confirmed coronavirus disease in the overall number of persons tested on a given day.

**Figure 5:**
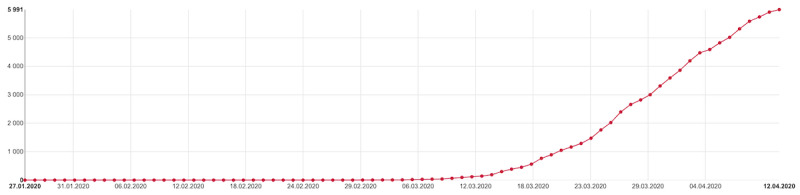
Overall (cumulative) number of persons with laboratory-confirmed coronavirus disease according to reports by regional public health authorities and laboratories.

In the stages of design and development of this web app, particular emphasis was placed on the character of presented data, which might be viewed by users anywhere and anytime. That was one of the reasons why responsive web design was among the main requirements, together with the overall optimization for mobile devices. The majority of line graphs and bar charts, therefore, primarily display an overview from the last 14 days, which is always adjusted even to small screens of mobile phones without the user having to manipulate the graph in any way. An additional complex view of the entire period of epidemic follow-up means that users of smaller devices may need to manipulate the graph if they want to display all values (Figures 4 and 5). A tabular overview is the last option available, displaying all values in rows and columns of a table. Other types of charts are also available on the website, such as the pie chart, stacked bar chart, or grouped bar chart, as well as map visualization and standard tables. Moreover, all published data are available to download in an open data format.

### Open Data Sets

Open data sets intended for further processing are an integral part of this information website. Source data in .CSV (comma-separated values) and .JSON formats are published daily at regular intervals, and can be downloaded by anyone who wants to process them either by a computer or manually. Data set schema are also included, describing the structure of records. Normalized schemas contain the list and definitions of all descriptive attributes as well as the technological representation of the data schema. All data sets published in this way are linked to records in the National Catalogue of Open Data (provided by the Ministry of Health of the Czech Republic), which is administered by the IHIS CR.

### Analysis of the Number of Visits

The Google Analytics component is used to monitor and to analyze the users’ behavior on the website. The objective of the development team was to monitor all basic characteristics such as the number of sessions, page views, ways of user acquisition (direct, organic, referral), type of device used (mobile, tablet, desktop), display resolution, and web browser. An overview of sessions on a given day and time makes it possible for the development team to plan timely releases of new versions of the portal and to update information. The website was officially launched on March 11, 2020, and has immediately become the primary communication channel employed by the health care sector to present the current situation regarding the COVID-19 epidemic. In the period between March 11, 2020, and April 12, 2020, the web portal registered 13,634,325 sessions and 19,662,351 page views. Returning visitors accounted for 16,286,551 (more than 80%) of users. This trend can also be confirmed by the graph showing how visitors landed on the website. A total of 7,158,020 (more than 50%) of visits were direct (ie, the website URL was typed into a browser) or were the result of an organic search (ie, users employed search engines such as Seznam.cz or Google.com). From the beginning, the development team designed the website as mobile-first. With regard to the portal contents, we assumed that its visitors would want to see the information fast and at any time. A total of 8,248,766 (more than 60%) of visitors used a mobile device or a tablet. A focus on graphs being drawn on displays with a certain resolution was no less important. The most usual width of mobile devices was 360 px, which comfortably accommodates a summary graph containing information from the last 14 days. Despite the clear preference of mobile devices, we also had to bear in mind that all graphs had to be well displayed in desktop browsers. Although Chrome was the most frequently used browser (8,112,423 of users, almost 60%), we also had to consider that some users prefer Internet Explorer (version 11.0 or lower), which accounted for more than 160,000 sessions. We used the Google Data Studio tool to create a dashboard that presents all needed information (see Multimedia Appendix 2) and thus provides a clear and easily available report. After the publication of open data sets and of a publicly available application programming interface on March 28, 2020, more than 100,000 page views were recorded over the next few days.

## Discussion

The web-based app introducing an overview of the current spread of COVID-19 in the Czech Republic has been designed, developed, and implemented in accordance with the CRISP-DM methodology. All interactive graphs, maps, and tables fully respect strict rules of data management in the health care sector, where data reporting, collection, processing, validation, analysis, evaluation, and final publishing are under the supervision of the Ministry of Health of the Czech Republic. The online interactive overview of the current spread of COVID-19 in the Czech Republic [21] provides comprehensive information to the general public in a well-arranged manner. Since the launch of the first version of the website, the development team has not only systematically collected and evaluated suggestions on improvements from the general public but has also responded to the needs of leadership in the health care sector and of the media. Selected requirements (ie, those that are not contrary to legislation on personal data protection and that do not lead to a direct identification of an individual) are subsequently implemented and released in the next version. At the beginning of March 2020, the epidemiological situation in the Czech Republic was deemed rather serious, so many measures have been put in place aiming to curb the epidemic on a nationwide level and as effectively as possible. This is why an entire family of web apps focusing on COVID-19 has been under development. In addition to the existing overview of the current situation in the Czech Republic, two more online systems are planned to be launched in a short time (ie, in the next few weeks): (1) an epidemiological portal on COVID-19, which will present the descriptive demography, mathematical prediction models, overviews of incidence, prevalence and mortality, the R coefficient, and other relevant information; and (2) an online control room for intensive care, containing the latest reporting on occupancy and availability of beds in real time, including an interface for a quick entry of the currently free capacity for patients with COVID-19 positive cases versus patients with COVID-19 negative cases. All three online tools have been primarily designed with the objective to keep everyone across the Czech Republic informed and to provide objective, data-based views for further decisions made by the leadership of the health care sector and by the emergency committee dealing with the COVID-19 epidemic in the Czech Republic.

## Appendix

Multimedia Appendix 1 Diagram of the cross-industry standard process for data mining reference model.

Multimedia Appendix 2 Selected visualization of coronavirus disease epidemiology in the Czech Republic. A: incidence of confirmed cases of coronavirus disease in the Czech Republic by region per 100,000 people. B: Overall number of persons who tested positive for coronavirus disease in the Czech Republic. C: Daily overview of the number of persons with newly confirmed coronavirus disease over the last 2 weeks. D: Overall number of persons with laboratory-confirmed coronavirus disease by age group.

Multimedia Appendix 3 Overview of monitored statistics regarding the number of visits (period: March 12-April 12, 2020).

## Abbreviations

COVID-19: coronavirus disease
CRISP-DM: cross-industry standard process for data mining
CSV: comma-separated values
IHIS CR: Institute of Health Information and Statistics of the Czech Republic
ISID: Information System of Infectious Diseases
JSON: JavaScript Object Notation
LAB: laboratories
NHIS: National Health Information System
PPE: personal protective equipment
RPHA: regional public health authority
SARS-CoV-2: severe acute respiratory syndrome coronavirus 2
