# Supplementary figures and images for: Complex Reporting of the COVID-19 Epidemic in the Czech Republic: Use of an Interactive Web-Based App in Practice
Source: J Med Internet Res. 2020 May 27;22(5):e19367. doi: 10.2196/19367 (PMC7254961; doi:10.2196/19367)

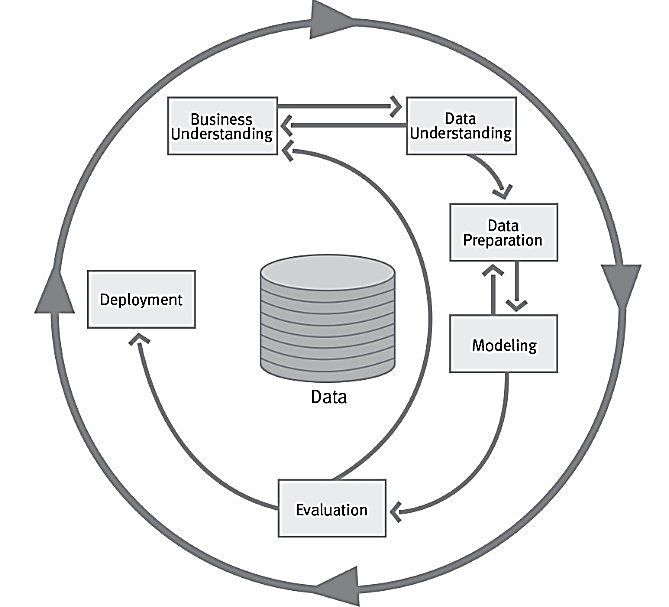

Supplement: Multimedia Appendix 1 [file jmir_v22i5e19367_app1.png]

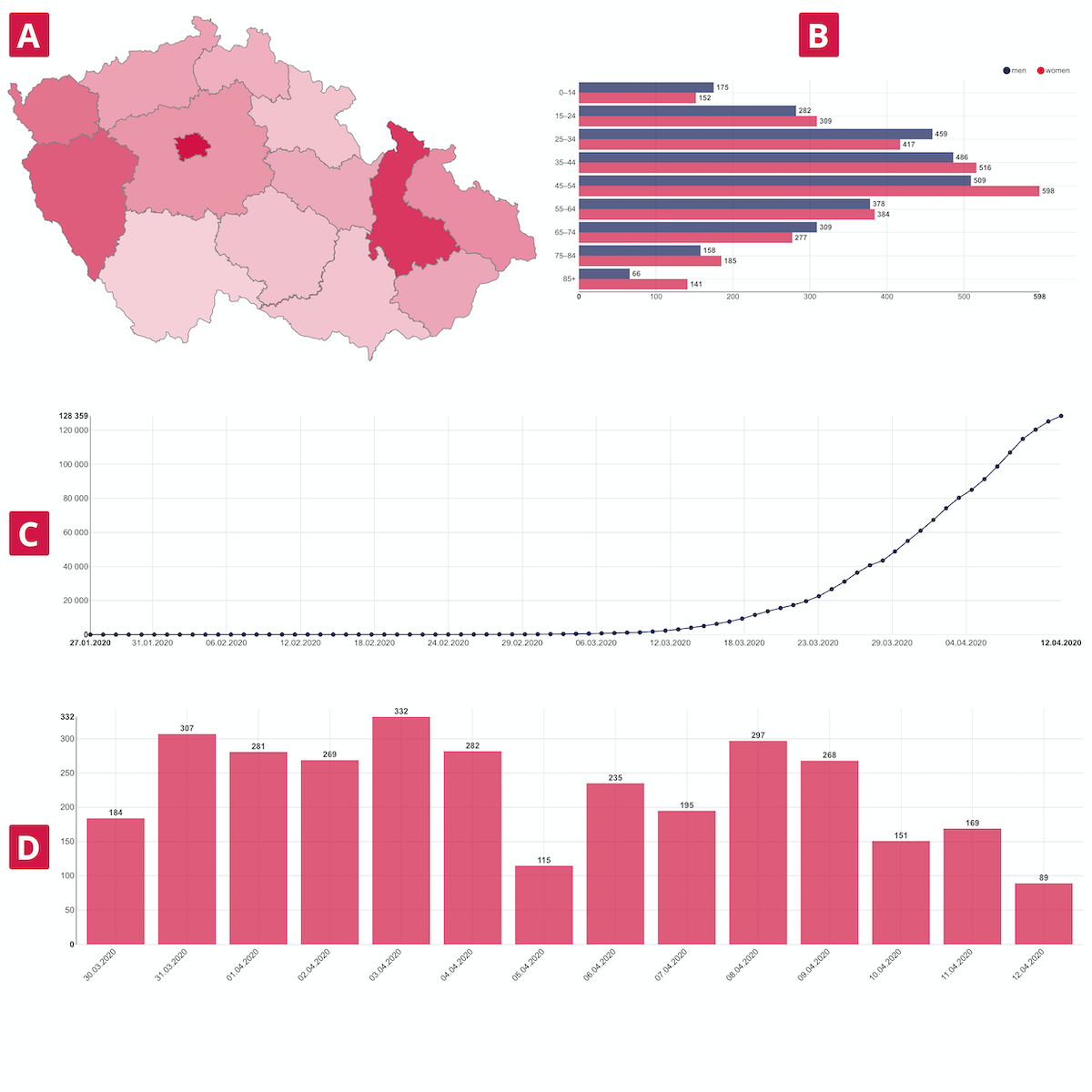

Supplement: Multimedia Appendix 2 [file jmir_v22i5e19367_app2.png]

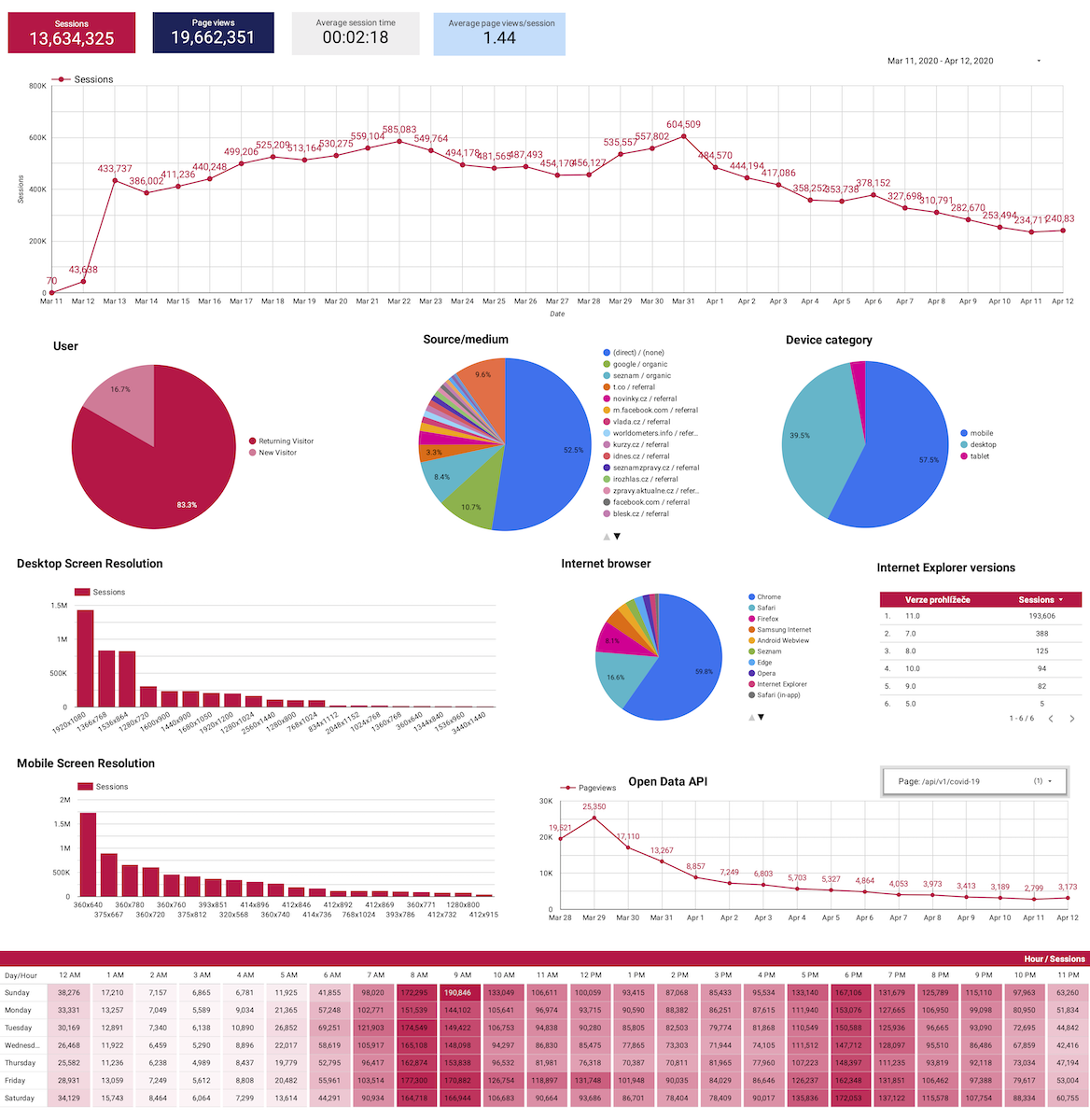

Supplement: Multimedia Appendix 3 [file jmir_v22i5e19367_app3.png]
